# Autism in Adulthood: Clinical and Demographic Characteristics of a Cohort of Five Hundred Persons with Autism Analyzed by a Novel Multistep Network Model

**DOI:** 10.3390/brainsci10070416

**Published:** 2020-07-01

**Authors:** Roberto Keller, Silvia Chieregato, Stefania Bari, Romina Castaldo, Filippo Rutto, Annalisa Chiocchetti, Umberto Dianzani

**Affiliations:** 1 Adult Autism Center, Mental Health Department, Health Unit ASL Città di Torino, 10138 Turin, Italy; rokel2003@libero.it (R.K.); chieregato.s@gmail.com (S.C.); stefania.bari@aslcittaditorino.it (S.B.); romina.castaldo@aslcittaditorino.it (R.C.); 2 Department of Psychology, University of Turin, 10100 Turin, Italy; filipporutto@gmail.com; 3 Department of Health Sciences, Universita’ del Piemonte Orientale, 28100 Novara, Italy; annalisa.chiocchetti@med.uniupo.it

**Keywords:** autism spectrum disorder, adulthood, diagnosis, intervention

## Abstract

Autism spectrum disorder (ASD) is a neurodevelopmental disorder characterized by deficits in communication and relational skills, associated with repetitive verbal and motor behaviors, restricted patterns of interest, need for a predictable and stable environment, and hypo- or hypersensitivity to sensory inputs. Due to the challenging diagnosis and the paucity of specific interventions, persons with autism (PWA) reaching the adult age often display a severe functional regression. In this scenario, the Regional Center for Autism in Adulthood in Turin seeks to develop a personalized rehabilitation and enablement program for PWA who received a diagnosis of autism in childhood/adolescence or for individuals with suspected adulthood ASD. This program is based on a Multistep Network Model involving PWA, family members, social workers, teachers, and clinicians. Our initial analysis of 500 PWA shows that delayed autism diagnosis and a lack of specific interventions at a young age are largely responsible for the creation of a “lost generation” of adults with ASD, now in dire need of effective psychosocial interventions. As PWA often present with psychopathological co-occurrences or challenging behaviors associated with lack of adequate communication and relational skills, interventions for such individuals should be mainly aimed to improve their self-reliance and social attitude. In particular, preparing PWA for employment, whenever possible, should be regarded as an essential part of the intervention program given the social value of work. Overall, our findings indicate that the development of public centers specialized in assisting and treating PWA can improve the accuracy of ASD diagnosis in adulthood and foster specific habilitative interventions aimed to improve the quality of life of both PWA and their families.

## 1. Introduction

Autism spectrum disorder (ASD) is a neurodevelopmental disorder with a prevalence ranging from 1% in the general population to ~1.9% in specific population groups [1,2]. ASD is typically characterized by deficits in socio-emotional reciprocity, impaired verbal and non-verbal communication skills, and an inability to develop and maintain adequate social relationships with peers, often associated with repetitive verbal and motor behaviors, restricted patterns of interest, need for a predictable and stable environment, and hypo- or hypersensitivity to sensory inputs. The onset of clinical symptoms occurs during the early years of life [1].

A large number of persons with autism (PWA) also meet criteria for co-occurring psychiatric conditions at a significantly higher rate compared to non-autistic populations [3,4]. Consequently, the socio-economic impact of ASD has become an increasing concern for public healthcare systems, which are now oriented toward an early diagnosis and intervention to improve ASD patient management while reducing the costs related to ASD care. However, there is paucity of guidelines for the diagnosis and management of PWA in adulthood. This aspect is of particular relevance in light of the detrimental effect that functional regression in these patients can exert on their psychosocial and work wellbeing. Indeed, PWA and their families are exposed to a “lifetime of difficult transitions” due to a limited number of service providers and resources alongside stringent and restrictive program funding criteria. As a result, there is widespread concern about the ability of some individuals with ASD to establish meaningful lives in adulthood [5].

Adolescents and adults with ASD are largely an underserved population. In a survey in Poland, the vast majority of parents of young PWA complained about the lack of assistance and support for PWA (93.5%) and the many difficulties in accessing the available services (82.7%) [6]. Indeed, low-income families and those living outside large cities have to deal more frequently with barriers to service access. This is particularly important in light of the fact that adult ASD patients need targeted psychosocial support to meet increasing social demands related to independent living, personal relationships, and successful employment. It is estimated that no more than 20% of adults with ASD have good or very good outcomes in these areas [7,8,9].

Studies from the US and Canada have reported that the number of outpatient services used by PWA decreases from childhood to adulthood and continues to decrease until late adulthood, while the use of medications and in-patient services increases [7,8,9], with very limited social integration, poor job prospects and high rates of mental health problems [10]. Indeed, the ensuing social isolation and unemployment further contribute to high rates of depression, anxiety, and other psychiatric disorders [11,12].

Qualitative studies have shown that transition outcomes among ASD patients are negatively affected by several factors such as poor person-environment fit, uncertainty about the roles of parents and the lack of comprehensive or integrated services. These findings have also revealed the aspects of familial, organizational and policy contexts that may be targeted for interventions. In this regard, stakeholders emphasize that supports should be individualized and focused on the changing aspects of the young adult’s social and physical environment rather than on behavioral changes [12,13,14]. Thus, there is widespread consensus that policymakers should address economic, regional, and age-related inequities in access to services among PWA [6,15]. Furthermore, adult-oriented healthcare services should work directly with PWA and their support networks to facilitate successful engagement with services and enable adults to manage their mental health needs [16].

To address these challenges, in 2009, a specialized Regional Center for Autism in Adulthood has been established at the department of Mental Health in Turin. The aim of the Center is to follow up PWA who received a diagnosis of autism in childhood/adolescence or individuals with suspected adulthood ASD, with the aim of improving the accuracy of ASD diagnosis and identifying a personalized rehabilitation and enablement program and this study describes the clinical and demographic characteristics of the participants in the survey outcomes of the diagnostic path.

## 2. Materials and Methods

Participants were adults referred to the Regional Center for Autism in Adulthood in Turin by general psychiatrists across the Piedmont region—the region has an adult population of 3,600,000 inhabitants—due to ASD or suspected autism, with the aim of designing a personalized intervention plan. Each participant was evaluated using a model developed by the center, namely the Multistep Network Model. This tool consists of a multistep diagnostic and evaluation assessment that combines diagnostic evaluation with a personalized life project devised by a team of psychiatrists and psychologists of the center, the PWA and their family members, social workers, members of the educational and vocational services (i.e., teachers and job trainers) and employment agencies (i.e., job-brokers). The main goal of the approach is to set up a network of integrated services to serve the needs of PWA in the Piedmont region.

All clinical evaluations, testing and treatments were conducted in the center upon written informed consent signed directly by the participants or their guardians, authorizing data collection and processing as well. Not requiring a specific authorization from the Ethics Committee during the course of daily clinical activity, all clinical and research activities described in this study were authorized by the Medical Director of the Local Health Unit DSM To2 in 2009. Such authorization was subsequently confirmed by the Director of the Local Health Unit Città di Torino in 2019. The research was also authorized by the Institutional Board of the Piedmont Public Health Agency by means of two Regional Council Resolutions (DGR) No. 22-7178, 3 March 2014 and No. 88-8997, 16 May 2019.

Multistep Network Model:

Step required for diagnosis and individualized project:

(a) First meeting with the parents: (i) careful collection of life history, (ii) all interventions carried out, (iii) needs and expectations—the direct meeting with the subject to be evaluated takes place only in the case of suspected high functioning PWA;
(b) Meeting with the PWA or individual with suspected autism: (i) welcoming and creating a human supporting relationship, (ii) clinical evaluation of the symptoms presented, (iii) clinical evaluation of any psychopathological symptom in co-occurrence, (iv) objective neurological evaluation, and (v) clinical evaluation of cognitive functioning;
(c) Assessment of the intellectual profile by using appropriate tests for the level of clinical functioning (Wechsler Adult Intelligence Scale IV edition; Raven; Leiter) and, if necessary, neuropsychological testing [17,18,19];
(d) Evaluation tests for suspected autism. The choice of tests to be performed is based on the clinical functioning and cognitive profile—Autism Diagnostic Interview-revised (ADIr) for all; Ritvo Autism and Asperger’s Diagnostic Scale-revised (RAADS) or Autism Diagnostic Observation Shedule-2nd Edition (ADOS 2) or Childhood Autism Rating Scale, Second Edition (CARS2-ST)depending on the level of functioning defined according to the clinical level and cognitive profile) [20,21,22,23];
(e) Evaluation of the adaptive functioning profile (e.g., Vineland/Adaptive Behavior Assessment System – Second Edition) [24,25]; Test evaluation of psychopathological functioning—if there is a clinical suspicion—with scales for intellectual functioning evaluation (Structured Clinical Interview, Minnesota Multiphasic Personality Inventory-2, Systematic Psychopathological Assessment for persons with Intellectual and Developmental Disabilities - General screening, Rorschach) [26];
(f) Medical evaluation focused on general health and specific conditions of neurodevelopment, including neuroimaging, genetic, metabolic evaluation, Electroencephalogram (EEG), depending on the individual’s situation;
(g) Network meetings between operators of the Center for Autism in Adulthood and the patient’s family members. When possible, there should also be meetings between the PWA and the operators involved in the clinical management of the patient, i.e., the child neuropsychiatrist, and if in transition age, social workers, school teachers, job trainers, and educators. In addition, all personal information and that related to the life context should be collected. These meetings should ultimately lead to the creation of a life project through the integration of clinical information and that derived from the care network, above all taking into account the preferences and wishes of the PWA and their families;
(h) The activation of a habilitative path provided directly by the center and/or presentation of the project to a Medico-Legal/Social Health Assessment Committee for evaluation of its appropriateness and allocation of the budget for the projects that will be delivered by accredited private healthcare centers.

## 3. Results

We performed a descriptive analysis of our cohort consisting of 500 participants.

The sample of participants is described in Table 1.

According to DSM5 criteria, ASD levels diagnosed in adult patients were as follows: level 1 in 39% (*n* = 193), level 2 in 27.1% (*n* = 135), level 3 in 18% (*n* = 90), neurodevelopmental disorder NOS not otherwise specified in 3% (*n* = 15), personality disorder (no autism) in 2.8% (*n* = 14) and psychosis (no autism) in 2.2% (*n* = 11). Additionally, 67.2% (*n* = 336) of participants displayed psychiatric and neurological co-occurrences, as shown in Table 2 and Table 3 [1].

Intellectual disability was found in 53% (*n* = 265), which was mild in 35.8% of cases (*n* = 95), moderate in 34% (*n* = 90), and severe in 30.2% (*n* = 80), while 11% of the sample (*n* = 55) had a speech disorder.

Concerning therapies, 42% (*n* = 207) received psychotropic drugs and 14% anti-epileptic drugs (*n* = 69). Relevant medical comorbidities were epilepsy (16.6%; *n* = 82), allergies (8.4%; *n* = 39), and gastrointestinal complaints (21.3%; *n* = 99).

The parent’s age at the time of birth ranged 17–48 years (mean ± SD: 30.72 ± 5.4) for the mothers, and 18–60 (mean ± SD: 34.21 ± 6.3) for the fathers; 15.5% of mothers experienced complications during pregnancy (*n* = 72) (Table 4). Moreover, 27.8% of the mothers had complications during delivery (*n* = 130).

With regard to neurodevelopmental stages, language skills on average appeared at 25.1 months (SD 19.94, range 6–180 months), and walking skills were achieved at 15.7 months (SD 6.3, range 8–60 months).

Table 5 reports the ages of PWA when the first symptoms became worrisome for the PWA’s family. The average age of symptom recognition was 41.6 months (SD 54.3), with the most delayed recognition occurring at the age of 50 years.

Symptoms at onset ranged from social-relational isolation in 38.1% of cases (*n* = 166), delay in the appearance of language in 25.7% (*n* = 112), inadequate relationship with peers in 15.1% (*n* = 66), stereotypies in 8.7% (*n* = 38), loss of language in 5.5% (*n* = 24), subjective experience of diversity (in individuals with high functioning autism in 3.2% (*n* = 14), and motor-coordination difficulties in 3.2% (*n* = 14).

First diagnosis of autism was made at the average age of 10.8 years (SD 12.3), but the diagnosis was revised in 49.4% of cases (*n* = 246), with an average of 26.5 years (SD 10.5). In 24.8% of cases (*n* = 123) the diagnosis was changed. Of note, during childhood only a small proportion of participants received medical assessment: 26.1% (*n* = 126) were evaluated with MRI (Magnetic resonance imaging) brain scan, 13.5% (*n* = 65) with cranial computed axial tomography, 16.8 (*n* = 81) with genetic analysis, and 16.2 (*n* = 78) with metabolic screening.

The types of intervention administered during childhood and adolescence are described in Table 6. No specific interventions were prescribed in 23.4% of cases (*n* = 117).

The analysis of schooling revealed that 6 out of 500 participants had an elementary education degree without completing middle school (1.2%), whereas 137 subjects (27.4%) finished middle school but did not pursue secondary education. Fifty-seven participants (11.4%) started a secondary education program but dropped out. Thus, 38.8% of participants earned a middle school diploma, while 52.2% of them completed successfully a secondary school program. Only 4.4% of subjects enrolled in a post-secondary program. Notably, 70.1% of participants (*n* = 337) needed additional teaching support at school, and 31.7% of subjects were bullied (*n* = 146). Unfortunately, bullying occurred in almost all situations related to Level 1 Autism (DSM5 criteria, ASD Requiring support) [1].

Neuropsychiatric disorders were reported in 53% of family members—familiarity up to III degree—*n* = 265. The most frequent disorders were depression (21%; *n* = 56), autism (16.2%; *n* = 43), psychosis (14%; *n* = 37), intellectual disability (13.2%; *n* = 35), dementia (5.3%; *n* = 14), Parkinson’s disease (4.2%; *n* = 11), specific learning disability (3.8%; *n* = 10), drug abuse 3.8% (*n* = 10) and Down syndrome (3.4%; *n* = 9).

After the evaluation, 189 out of 500 people (37.8%) received interventions directly delivered by the Center for Autism in Adulthood. Specifically, study participants were administered social skills interventions (37%; *n* = 69), cognitive-behavioral psychotherapy (26%; *n* = 50), expressiveness through visual art (9%; *n* = 17), parenting support group programs (9%; *n* = 17), body-vocal expressiveness (7%; *n* = 13), cognitive enhancement (Feuerstein method) (6%; *n* = 12), neuropsychological rehabilitation (3%; *n* = 6), habilitative intervention with animals (2%; *n* = 3) and musical expressiveness (1%; *n* = 2).

Indirect interventions were provided by accredited private centers as follows: 50.4% of patients (*n* = 143) attended a daily center, 31% (*n* = 88) underwent psychoeducational intervention and 7.4% (*n* = 21) followed a work-oriented training path. From the psychiatric standpoint, 6% (*n* = 17) were assisted by a mental health center due to the presence of severe psychopathological co-occurrences.

Finally, analyzing the housing situation, most PWA still lived with their families, with only 8.1% (*n* = 40) living in a residential structure offering around-the-clock assistance. Only 1% (*n* = 5) shared an apartment with few other disabled people, whereas 11% (*n* = 54) lived independently.

## 4. Discussion

Autism in adulthood is a complex condition that should be distinguished from ASD in childhood and adolescence, especially for high level of co-occurrence and specific needs [9,27,28]. In this regard, there are several reasons that prompted us to develop a new integrated assessment models, termed the Multistep Network Model, able to improve the accuracy of autism diagnosis in adults, a growing concern for public healthcare systems, with the ultimate goal of developing personalized rehabilitation and enablement programs. Firstly, there are a number of undiagnosed PWA, especially among women with ASD, that need to be detected [29,30,31]. Secondly, the transition to adulthood needs to be built by means of a habilitative program specific for adult (personalized life-project). Thirdly, we can clinically observe high rates of psychiatric comorbidities, requiring specific treatment. Finally, as caregivers become older, there is a marked decrease in family resources, which translates into a higher level of concern for the future of PWA [9,27].

Currently, PWA can follow a wide range of pathways during their transition to adulthood, such as attending college, entering the labor force, and achieving a degree of independent living. Less cognitively able individuals, on the other hand, may be eligible for state benefits or may access supported employment programs. Thus, clinicians need to familiarize with the unique needs of adults with ASD to be able to administer specific supports and interventions to these patients to ensure their best possible social integration in the community [28].

Among high functioning PWA, our study shows that these patients, especially women, are more likely to experience feeling different. This awareness begins at an early age—“I have always felt different from others” is a frequently used expression among adults with autism—, suggesting a more internalized autism syndrome [31]. Considering the possibility to decrease the risk of ASD, we can observe that prenatal, perinatal, and postnatal factors have been associated with autism [32]. The average age of parents at the time of birth, which could have increased the genetic risk in our sample, is in line with that of the general population—i.e., 30 years for the mother and 34 years for the father in PWA vs. 30.6 years for the mother and 34.2 years for the father in the general population. The role of pregnancy and delivery with respect to the pathogenesis appears to be relevant as 15.5% of PWA had complicated pregnancies and 27.8% had complications at delivery. These elements may therefore have been decisive for the development of hypoxic brain injuries with neuronal damages. According to a meta-analysis by Wang et al., during the prenatal period, the factors associated with autism risk were maternal and paternal age ≥ 35 years, mother’s and father’s ethnicity (i.e., Caucasian and Asian), gestational hypertension, gestational diabetes, maternal and paternal education level (i.e., college graduation), threatened abortion and antepartum hemorrhage. During the perinatal period, the factors associated with autism risk were caesarian delivery, gestational age ≤ 36 weeks, parity ≥ 4, spontaneous labor, induced labor, no labor, breech presentation, preeclampsia, and fetal distress. During the postnatal period, the factors associated with autism risk were low birth weight, postpartum hemorrhage, male gender, and brain anomaly [32]. Thus, health policy programs aimed at improving pre-natal and peri-natal conditions, including environmental factors that may preclude a safe pregnancy, may play an important role in decreasing ASD incidence.

Therefore, the implementation of training programs for teachers of nursery and primary schools together with routine screening by pediatricians is highly recommended to improve ASD diagnosis at an early age.

It is also important to point out that language problems in ASD patients are not only related to onset delay but also to semantic deficits: children with ASD produced more global, rather than local, semantic features in their definitions than children with normal language. An over-reliance on global, rather than local, features in children with ASD may reflect in-depth deficits of word knowledge [33].

The need for a diagnostic revaluation over time during the transition to adulthood is clearly documented by the fact that almost half of the PWA (49.4%) required revision of diagnosis—mostly due to changes of the clinical picture, nosographic reference parameters, cognitive functioning, psychopathological co-occurrences, etc.—, which was modified in 24.8% of PWA. The revaluation is particularly useful in detecting not only modifications of cognitive level of functioning but also the co-occurrence of a psychiatric disorder. Consistently, ASD diagnoses were confirmed in all PWA if made previously. Co-occurring mental health conditions are more prevalent in ASD than in the general population and a careful assessment of mental health is an essential component of care for all PWA and should be integrated into clinical practice with specific assessment [34,35,36,37,38]. Given that the co-occurrence of personality disorders is typically related to young adulthood but not childhood, the assessment should also explore personality in ASD. Psychopathological co-occurrences were present in 67.2% of the sample. They included the high prevalence of challenging behavior/problem behavior, which constitutes one of the main issues in family management. For this purpose, adequate diagnostic methods should be implemented, aimed primarily at excluding organic causes, with a subsequent activation of response programs, possibly behavioral, limiting psychopharmacological intervention as much as possible. The co-occurrence of Attention Deficit Hyperactivity Disorder (ADHD, 9.5%) is likely underestimated because of difficulty in making ADHD diagnosis in severe cases of autism and the presence of overlapping symptoms in both disorders. However, these should be regarded as continuous neurodevelopmental disorders instead of categorical comorbidities [39]. Generally, obsessive-compulsive disorders (OCD, 6.8%) are difficult to diagnose in PWA due to the presence of autistic rituals that, even though they may seem obsessive, represent an epiphenomenon of the primary disorder [40]. Major depressive disorder (6.3%) has a lower prevalence in our sample than what reported in the literature. This may be due to an increase in diagnostic performance that allowed us to rule out major depression forms belonging to other nosographic pictures. However, we did find a co-occurrence of psychosis and paranoid personality disorder in 5.7% of cases: psychotic forms were represented by both reactive forms with paranoid characteristics, often activated by triggers, such as bullyism and mobbing, and an interpretative deficit of reality, alongside rare forms of the schizophrenic type [41]. The latter is probably ascribable to a common genetic load with respect to the neurodevelopmental disorder, which expresses a first autistic phase and a second phase of schizophrenic deterioration [42,43,44]. Concerning the structuring of a paranoid personality disorder, we identified several situations fueling a chronic persecutory reading of the external human environment, such as the inability to read the surrounding environment, experience of exclusion, distress originated from bullying and mobbing episodes, and problems with social and work insertion. Use of psychotropic drugs is reported in 42% of the sample, and antiepileptics in 14%, with the presence of comorbidity for epilepsy in 16.6% of subjects. In a large cohort in the UK, approximately one-third of the identified cohort was prescribed at least one psychotropic medication and the prevalence increased 3.3-fold from 0.109 per 100 persons in 2009 to 0.355 per 100 persons [45]. More recently, a survey carried out in the Emilia Romagna region in Italy showed that 74.5% of adults with ASD were being treated with a psychotropic drug, with 41.6% of the participants using an antipsychotic drug, 15.19% two or more antipsychotic drugs and 5.17% a long-acting antipsychotic drug [46]. This large increase in psychotropic drug use among PWA calls for strict monitoring of drug prescriptions by healthcare services. Regarding psychopathology, in particular neurodevelopmental disorders, we found that a high proportion (53%) of family members of PWA (up to the third degree) report neuropsychiatric disorders, with one family member out of five expressing a depressive disorder, partly reactive to chronic stress and partly independent. With respect to neurodevelopmental disorders, 16% of family members display forms of autism—therefore, at least 16 folds more frequently than the general population—, while others show intellectual disability, specific learning disorders, Down syndrome, ADHD, stereotypes, with a cumulative percentage for neurodevelopment disorders reaching 39.6%. These data underscore the importance of providing genetic-metabolic evaluation also in adulthood PWA, since a transgenerational transmission of the vulnerability to neurodevelopmental disorders is highly likely [47,48]. Psychotic disorders were reported in 14% of family members, raising a question with respect to common biological/genetic factors shared between autism spectrum disorders and psychotic disorders [49]. Another interesting observation regards the high incidence of neurodegenerative disorders, such as dementia—Parkinson’s disease is present in 4.2% of the participants—which warrants further in-depth studies aimed to elucidate its potential pathogenetic ramifications.

At the somatic level, the most evident finding is the lower prevalence of allergies in PWA compared to that of the general population (8.4% vs. ~30%, respectively), indicating substantial differences in the immune system response to allergens between the two groups. Furthermore, we recorded gastrointestinal disorders in 21.3% of cases, which are often the basis of behavioral disturbances, especially in seriously affected PWA experiencing difficulties in communicating. Medical evaluation plays a key role in ASD, especially during the developmental age, since it can rule out secondary forms of autism that could be specifically treated. Metabolic and genetic evaluations were observed in 6.2% and 16.8% of PWA, respectively. This may be the result of both the scarce attention paid to the organic aspects of autism and the attribution of autism pathogenesis to poor parental relationships in that historical period [50]. Consequently, even the interventions that PWA received during their developmental age often lacked specificity. In our sample, we recorded the following types of intervention: psychotherapy—rarely cognitive behavioral and more frequently psychoanalytic—in 29.9% of cases, speech therapy in 41.1%, psychomotor skill training in 38.6%, and non-specific psychoeducational interventions in 24.6%. In this context, speech and psychomotor therapies represented for years the standardized response offered by public services to autistic or disabled children.

A relevant finding in this study is represented by the high levels of co-occurrence of intellectual disability in more than a half of PWA. This percentage is seemingly higher than that recently reported by a study on childhood autism. If confirmed, this co-occurrence may be ascribable to the inadequate treatments received by PWA during their childhood, which presumably contributed to worsen their cognitive and functional abilities. This percentage may also indicate a general improvement in the diagnosis of high functioning forms of autism, which probably went undiagnosed in the past, especially in females [3].

Bullying is a trigger for psychopathology in adolescence and adulthood. One-third (31.7%) of PWA reported to have been bullied. This occurred to almost all PWA with the best functioning level (ASD level 1, DSM 5), which appear to be at high risk of psycho-traumatic events especially if undiagnosed. In keeping with other studies, autism severity did not significantly predict bullying-related behaviors. Consequently, secondary psychopathological comorbidity (e.g., anxiety, depression, etc.) may arise. Furthermore, this represents the level of autism in which the diagnostic evaluation is often lacking, as shown by those children mistaken for “little geniuses”, whose socio-relational deficit is seen as a side effect of academic excellence [51,52]. With regard to the severity of the ASD, since we are a public health assessment center, our PWA sample included all forms of autism, with 39% of minor forms, i.e., ASD level 1 DSM 5, including previously defined forms of high-functioning autism and Asperger’s syndrome—27.1% of level 2 autism (DSM 5), and 18% of severe forms of autism (ASD level 3 DSM 5) [1]. A small proportion included participants with neurodevelopmental disorders but with no sufficient information to categorize them in a specific subtype. The lack of information was due, for example, to the old age of PWA, which made it extremely difficult to gain information about his/her childhood from the parents. The few participants that did not meet the diagnostic criteria for autism were found to match those related to personality disorders (2.8%) and psychosis (2.2%), thus providing a bona fide of the assessment carried out by the Regional Center for Autism in Adulthood and the screening made by the general psychiatrists. Our organization is in fact based on a screening performed by a general psychiatrist to detect relevant psychiatric disorders before sending the patients to the Center. This triage policy has allowed us to save a considerable amount of clinical resources that would have otherwise been used to assess non-ASD patients. Our results also point out that the transition from childhood to adulthood in PWA represents a critical step for their families, whose management requires an overall re-evaluation, both at the clinical and project level. Consistently, the clinical evaluation model applied in this study is based not just on a process of progressive and deeper evaluation of the PWA or people with suspected autism and their families, but also on the available network of services that may be involved to formulate a personalized life-project (multistep network model). This personalized project is based on the information collected during the evaluation and may be delivered by the Regional Center for Autism in Adulthood or accredited private social centers. In the latter case, the project would need to be evaluated by a commission composed by members of the Health Public Agency and Public Social Services of the municipality of residence, which verifies the validity of the project and allocates a budget to support the external project. The interventions delivered directly by the Regional Center for Autism in Adulthood included paths for the improvement of social skills (social skill training) and cognitive-behavioral therapy. Group or individual support paths were activated for parents upon request. Another area of intervention concerned the improvement of expression and relationship skills, with activities aimed at improving communication through figurative arts—particularly for people with verbal communication impairment—and vocal-bodily expressive activities, using theater-based techniques. A subgroup of PWA, with medium-level autistic functioning and cognitive difficulties, followed a path specifically dedicated to improving cognitive functioning through structured learning techniques, such as Feuerstein or neuropsychological rehabilitation. Only in specific and selected exceptional situations, in the presence of severe communication deficits, interventions included the support of animals (three participants included) or communication through music (two participants). By analyzing the interventions provided indirectly by the accredited private partner, most PWA attended an educational activity covering most of the day and organized in groups (daily center; 50.4%), offering educational qualification courses and laboratories. In 31% of the participants, the activated path was instead based on individual educational activities or performed in small groups, delivering projects targeting specific skills of individual autonomy. For 7.4% of PWA, pre-work paths were activated: these consisted of training courses dedicated to PWA, lasting one to two years, made up of a theoretical part delivered in a classroom and a practical part via company internships. This type of path was dedicated to PWA with real job placement opportunities, thus needing less support, and managed by a professional school with teachers trained on autism and specifically dedicated to this type of course. In addition, the courses were prescribed provided that the PWA had followed a clinical path to improve and test his/her social skills and abilities to avoid stress deriving from real-life failures. Indeed, a person with autism needs to be adequately trained before being exposed to potentially harmful situations [53,54]. A small percentage of PWA (6%) was also followed by a Mental Health Center because these patients displayed a level of severity of the psychopathological comorbidity requiring a second parallel intervention dedicated to its management. This usually took place in outpatient clinics or day hospitals, but it may sometime require admission to the psychiatric ward. With regard to the housing situation, most PWA lived with their family of origin, whereas a small fraction (8%) lived in a community for disabled people with round-the-clock assistance. Only 1% of PWA resided in an apartment with other disabled people, while 11% achieved enough skills to live an independent life. Based on our findings, we propose a model for ASD in adulthood where public health and private services are integrated, and where the Regional Center for Autism in Adulthood plays a key role in managing PWA patients thanks to a joint effort between the private and public health sectors.

## 5. Conclusions

Among the several important aspects highlighted by our PWA analysis, the late age of autism diagnosis and the non-specific interventions provided during the developmental age are of particular relevance. As the current adult generation of PWA is apparently “lost”, it urgently needs a very complex intervention program [34]. Indeed, these are PWA who have often developed other psychopathological co-occurrences or manifest a challenging behavior/problem behavior usually associated with lack of adequate communication and relational skills, never taught to them during their educational path.

Altogether, our findings call for a radical improvement of the accuracy and timeliness of the diagnosis of ASD in adulthood and the implementation of specific interventions in favor of this often-neglected population. Such interventions should address as much as possible the autonomy and communication dimensions, especially in those individuals that did not received any specific interventions during their developmental age. Also, preparing PWA for employment is of the utmost importance, if possible, given the social value of work. In conclusion, the implementation of public centers dedicated to PWA represents a viable solution for improving the diagnosis of ASD in adulthood and ameliorating the quality of life of PWA and their families through specific habilitative interventions. These interventions, in accordance with the Autism Guidelines, should merge multidisciplinary professional teams, formed by psychiatrists, clinical psychologists, educators and rehabilitation therapists, with other entities, such as social services, schools and job agencies, all forming a network capable of assisting autistic children and adults along their social integration path. In light of hypotheses regarding developmental and environmental influences on the course of ASD, future studies should compare the changes in symptoms and presentation across adulthood to those occurring in childhood.

## Figures and Tables

**Table 1:** Description of the participants.

**Sample**	***n***	**%**	
Male	388	77.5	
Female	112	22.5	
**Age**	M = 31.7	SD = 10.7	Range: 18–82 years
**Place of Residence**	***n***	**%**	
Turin	262	52.9	
(Rural) Canavese Area	34	6.9	
Nichelino/Moncalieri and Carmagnola (Periurban areas)	55	11.1	
**Medico-Legal Assessment of Professional Competences**	***n***	**%**	
	302	65.4	
**In Details**			
46% civil disability level (CD)		2.1	
67% civil disability level (CD)		0.9	
75% civil disability level (CD)		7.1	

**Table 2:** Psychopathological and neurological co-occurrence in the study participants with autism.

Co-Occurrence	*n*	%
personality disorders	79	24%
challenging/problem behaviour	66	19.6%
Attention Deficit Hyperactivity Disorder (ADHD)	32	9.5%
epilepsy	23	6.8%
obsessive compulsive disorder	23	6.8%
major depressive disorder	21	6.3%
psychosis	19	5.7%
bipolar disorder	8	2.4%
anxiety disorder	6	1.8%
specific learning disorder.	6	1.8%
tic disorder	6	1.8%
oppositional defiant disorder	5	1.5%
deafness	5	1.5%
Down syndrome	5	1.5%
social phobia	5	1.5%
Tourette syndrome	5	1.5%
eating disorder	4	1.2%
blindness	3	0.9%
movement disorder	3	0.9%
substance abuse disorder	3	0.9%
dyspraxia	2	0.6%
language disorder	2	0.6%
X fragile syndrome	2	0.6%
tuberous sclerosis	1	0.3%
Turner syndrome	1	0.3%
XXY syndrome	1	0.3%

**Table 3:** Personality disorders in the study participants with autism.

Personality Disorders Co-Occurrence in ASD (PD)	*n*	%
paranoid PD	19	5.7%
borderline PD	18	5.4%
personality disorder not otherwise specified	7	2.1%
schizotypical PD	7	2.1%
avoidant PD	6	1.8%
obsessive PD	6	1.8%
narcissistic PD	5	1.5%
schizoid PD	5	1.5%
aggressive passive PD	3	0.9%
histrionic PD	2	0.6%
dependant PD	1	0.3%
**Total**	**79**	**24%**

**Table 4:** Prenatal and perinatal complications.

Complications	*n*	%	Note
During pregnancy	72	15.5	including 5.8% risk of abortion (*n* = 27)
Gestosis	12	2.5	
Twin pregnancy	7	1.5	
Depressive disorder	3	0.6	
Diabetes	1	0.2	
Thyroid disorder	1	0.2	
Toxoplasmosis	2	0.4	

**Table 5:** Age of PWA when the first ASD symptoms worried the family.

The Age When Symptoms Worried the Family	% PWA
6 Months	3.8
12 Months	11.3
18 Months	19.5
24 Months	42.6
30 Months	47.2
42 Months	80.3
48 Months	84.3
60 Months	97.8
72 Months	93.8

**Table 6:** Intervention administered during childhood and adolescence.

Intervention Type	*n*	%
Psychotherapy	143	29.9
Speech Therapy	200	42.1
Educational Interventions	117	24.6
Psychomotor skills Treatment	183	38.6
Delacato method	4	0.8
Portage method	13	2.7
